# TNF-α signaling: TACE inhibition to put out the burning heart

**DOI:** 10.1371/journal.pbio.3001037

**Published:** 2020-12-09

**Authors:** Gesine M. Dittrich, Joerg Heineke

**Affiliations:** Department of Cardiovascular Physiology, European Center for Angioscience (ECAS), Medical Faculty Mannheim, Heidelberg University, Mannheim, Germany

## Abstract

More than 20 years ago, Seta and colleagues hypothesized that cytokines, which are activated by myocardial injury, significantly drive heart failure progression and would therefore be effective targets to treat cardiac dysfunction. Unfortunately, several clinical trials inhibiting key cytokines like tumor necrosis factor alpha (TNF-α) and interleukin 1 beta (Il-1β) turned out negative or even revealed adverse clinical effects. Providing a potential mechanistic explanation for the ineffectiveness of TNF-α blockade in heart failure, novel findings demonstrate that the membrane-bound precursor form of TNF-α, transmembrane TNF-α (tmTNF-α), mediates cardioprotective effects during pressure overload-induced cardiac remodeling. This study suggests that preventing tmTNF-α cleavage by targeting the TNF-α converting enzyme (TACE) rather than inhibiting TNF-α signaling altogether might be a valuable therapeutic approach.

## Abstract 2

The Yin and Yang of TNF-α signaling in heart failure: this Primer explores the history of failure of TNF-α antagonistic therapy in heart failure and how a recent study suggests that inhibition of TNF-α converting enzyme TACE might be a way out of this dilemma.

Acute or chronic injuries of the heart initiate cellular signaling cascades causing a variety of adaptive and maladaptive processes [1]. Enhanced cardiac growth, based on hypertrophy of cardiac myocytes, for example, is an integral part of myocardial remodeling in response to injury and predisposes to heart failure. Besides activation of the renin-angiotensin-aldosterone system (RAAS) and desensitization of adrenergic signaling receptors, the increased synthesis and secretion of pro-inflammatory cytokines is a major hallmark of heart failure development. While therapeutic suppression of RAAS activation and β-adrenoceptors have proven to significantly combat heart failure progression, multiple clinical trials using anti-inflammatory approaches have failed to demonstrate positive effects or have even worsened heart failure. For example, treatment with tumor necrosis factor alpha (TNF-α) neutralizing drugs (Infliximab and Etanercept) had no beneficial effects on heart failure hospitalization or mortality, and treatment with high doses of Infliximab even caused adverse clinical outcomes [2,3]. As disappointing as these results were, they drew attention back to the underlying biological processes and led to further investigation of the molecular interplay between inflammatory mediators and cardiac remodeling.

While several preclinical studies have shown a direct negative effect of inflammatory cytokines on cardiomyocyte contractility, hypertrophy, and survival, answering the question whether inflammatory cytokines directly cause heart failure progression in an in vivo setup is certainly more difficult [4,5]. Although the administration of TNF-α via osmotic pumps was sufficient to promote cardiomyocyte hypertrophy and left-ventricular dysfunction [6], this most likely did not completely represent the complex inflammatory reaction during disease. Additional supporting data were provided by population studies, which found a 2-fold increased risk of developing heart failure when suffering from rheumatoid arthritis, a disease that is associated with a severe and prolonged systemic increase of inflammatory cytokines [7]. The “cytokine hypothesis” is therefore well supported by experimental and clinical data [8]. However, there is also evidence that inflammatory signaling is protective under certain conditions: After acute myocardial infarction, cytokines are up-regulated and orchestrate the resolution of cellular debris together with invading inflammatory cells. The elevated myocardial cytokines also signal to local fibroblasts, keeping them initially in a proteolytic state and preventing a premature differentiation into secretory and contractile myofibroblasts [9]. It is therefore very likely that therapeutic inhibition of the inflammatory reaction will only have beneficial effects if it outlasts the original stimulus.

The pro-inflammatory cytokine TNF-α signals through 2 different cell surface receptors, tumor necrosis factor receptor 1 (TNFR1) and tumor necrosis factor receptor 2 (TNFR2). Immunofluorescence stainings of uninjured human myocardium revealed localization of TNFR1 predominantly on cardiomyocytes and vascular endothelial cells and rarely on fibroblasts and leukocytes, while TNFR2 was mostly found on vascular endothelial cells and some leukocytes, but not on cardiomyocytes or fibroblasts. However, during inflammation, the mRNA expression and protein levels of TNFR2 in cardiomyocytes were strongly up-regulated. Production of TNF-α in the heart was restricted to microvessels and leukocytes [10]. Upon ligand binding, the adapter protein TNF receptor-associated DD (TRADD) is recruited to the intracellular death domain (DD) of TNFR1, which subsequently initiates downstream signaling that leads to rapid activation of nuclear factor kappa B (NF-κB) (Fig 1). While initial NF-κB stimulation is antiapoptotic, the release of TRADD and the receptor interacting protein (RIP) from the TNFR1 DD enables the formation of a cytoplasmic complex with the Fas-associated DD protein (FADD) and caspase 8, which results in cellular apoptosis. Unlike TNFR1, the intracellular part of TNFR2 does not contain a DD, and upon receptor activation, the TNF receptor associate factor-2 (TRAF2) is recruited to the intracellular TNFR2 receptor domain, where it associates with the TNF receptor associate factor-1 (TRAF1) and the cellular inhibitors of apoptosis 1 and 2 (cIAP1/2). Further downstream, the signaling of TNFR2 is mediated either via NF-κB inducing kinase (NIK) that activates IκB kinase (IKK), or through phosphatidylinositol 3-kinase (PI3K) activation of protein kinase B (Akt). Both TNFR2 pathways result in a persistent up-regulation of NF-κB, mediating the transcription of antiapoptotic genes and cellular protection [11].

**Fig 1:**
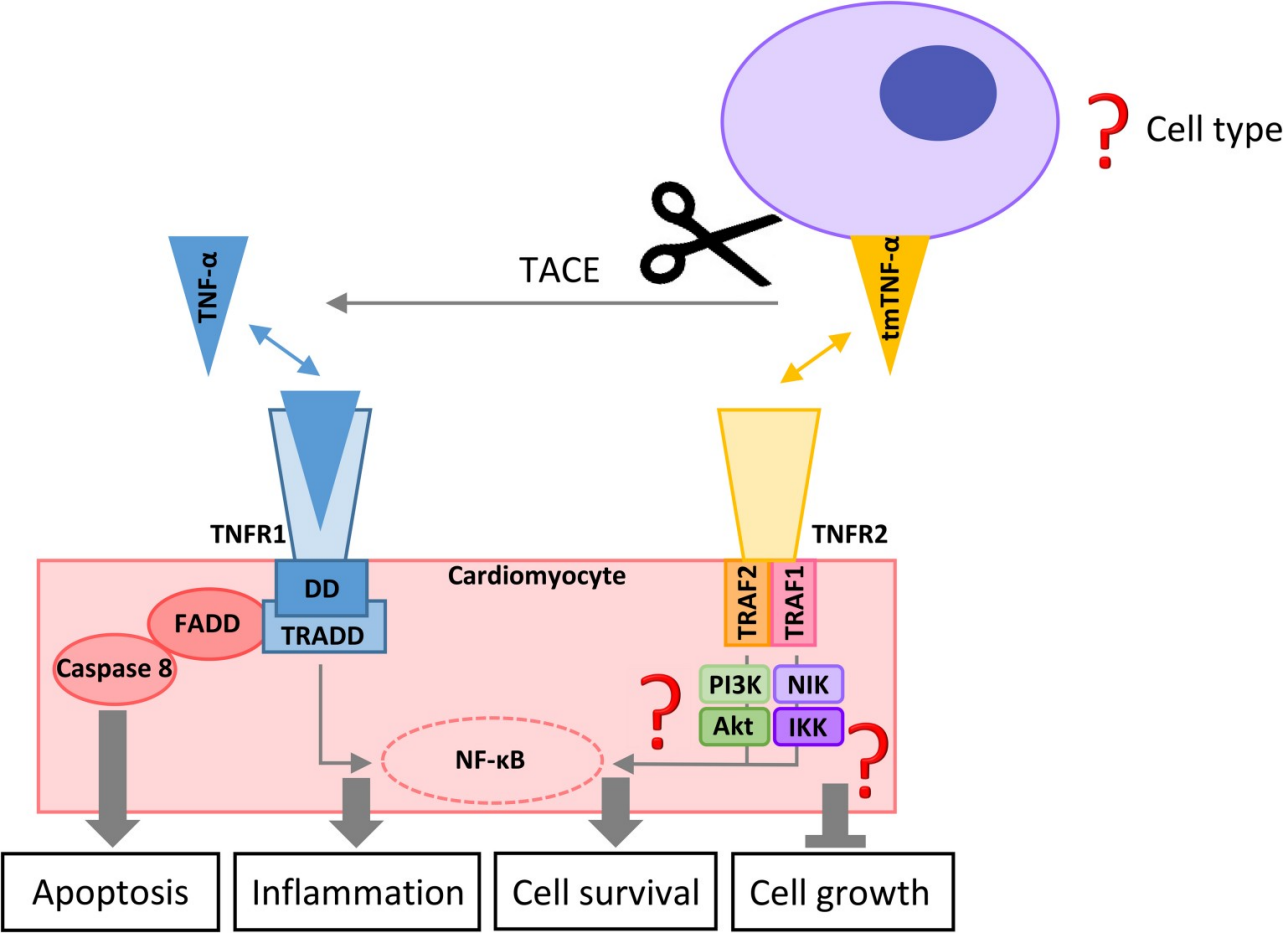
Schematic illustration of cardiomyocyte TNFR1 and TNFR2 signalings. Akt, protein kinase B; DD, death domain; FADD, Fas-associated DD; IKK, IκB kinase; NF-κB, nuclear factor kappa B; NIK, NF-κB inducing kinase; PI3K, phosphatidylinositol 3-kinase; TACE, tumor necrosis factor alpha converting enzyme; tmTNF-α, transmembrane tumor necrosis factor-α; TNFR1, tumor necrosis factor receptor 1; TNFR2, tumor necrosis factor receptor 2; TNF-α, tumor necrosis factor alpha; TRADD, tumor necrosis factor receptor-associated DD; TRAF1, tumor necrosis factor receptor associate factor-1; TRAF2, tumor necrosis factor receptor associate factor-2.

In the issued study, the authors investigated the effect of TNF-α signaling on pressure overload-induced cardiac hypertrophy [12]. Using mouse models to specifically delete the 2 TNF-α receptors, TNFR1 and TNFR2, they were able to examine their particular effects on cardiomyocytes and cardiac function. Interestingly, they found that only knocking out TNFR1 had beneficial effects on cardiac remodeling, while deletion of the TNFR2 even exacerbated the pressure overload-induced cardiac hypertrophy and dysfunction. Moreover, they demonstrate preferential binding of the insoluble transmembrane TNF-α (tmTNF-α) to the TNFR2, which they suggest as the primary mediator of protective TNF-signaling during cardiac remodeling. They further substantiated this finding by direct exposure of cultured cardiomyocytes to insoluble tmTNF-α ligand (expressed on NIH3T3 cells, a murine embryo fibroblast cell line), which protected them from stress-induced hypertrophy. Importantly, the anti-hypertrophic effects of tmTNF-α on cardiomyocytes were completely dependent on the presence of TNFR2, but not TNFR1.

While previous studies had already indicated a protective role for TNFR2-mediated signaling, whereas negative effects were predominantly associated with activation of TNFR1, the specific ligands for the receptors in the heart were previously less clear [13]. The current study poses an intriguing mechanistic explanation for the deleterious effects of anti-TNF-α treatments on heart failure; while antagonism to the soluble TNF-α would be desirable, since it indeed mediates cardiomyocyte hypertrophy and triggers further expression of the pro-inflammatory cytokines interleukin 1 beta (IL-1β) and interleukin 6 (Il-6), the blockade of the protective membrane-bound form tmTNF-α would disrupt its beneficial effects. Conversion of tmTNF-α into the soluble form is catalyzed by the TNF-α converting enzyme (TACE). Using an elegant approach, the authors further demonstrated that the pharmacological suppression of TACE activity improved cardiac remodeling and heart failure in mice during pressure overload.

Before these interesting new findings could be considered for clinical application, further questions are still open that will need to be investigated. One critical point to address is which cell type serves as primary provider of tmTNF-α and whether cardiac fibroblasts, endothelial cells, or leukocytes thereby contribute to the termination of soluble TNF-α–triggered cardiac tissue inflammation. Moreover, it is necessary to clarify whether TNF receptor expression on other cardiac cell types (apart from cardiomyocytes) might also (at least partially) mediate the effects of TNF-α in the heart. Studies with cell type–specific knockout of TNFR1 and TNFR2 or TNF-α would therefore be very interesting in the context of pressure overload or myocardial infarction. Additionally, the downstream signaling of TNFR2 requires further attention in order to understand its protective signaling response. It is so far unclear how TNFR2-mediated activation of Akt protects the heart from pressure overload or isoproterenol-induced hypertrophy, as Akt is well known to promote adaptive, but, upon prolonged activation, also maladaptive cardiomyocyte growth. Which other pathways become activated in cardiomyocytes upon TNFR2 activation? Investigation of isolated TNFR1 knockout cardiomyocytes after stimulation with tmTNF-α in a phospho-proteomics screen could, for instance, elucidate potentially protective signaling pathways. Moreover, the role of TACE deserves additional investigation, as this might be a potentially promising therapeutic target. Which cells are mainly mediating TNF-α cleavage by delivering TACE? What are the (transcriptional) mechanisms that induce its expression upon mechanical loading? Is it also induced in response to inflammatory stimuli? Answering these questions might open additional options for therapeutic intervention to prevent excessive TNF-α signaling without abolishing the protective effects of tmTNF-α on cardiac remodeling.

## Abbreviations

Akt: protein kinase B
cIAP: cellular inhibitors of apoptosis
DD: death domain
FADD: Fas-associated DD
IKK: IκB kinase
Il-1β: interleukin 1 beta
Il6: interleukin 6
NF-κB: nuclear factor kappa B
NIK: NF-κB inducing kinase
PI3K: phosphatidylinositol 3-kinase
RAAS: renin-angiotensin-aldosterone system
RIP: receptor interacting protein
TACE: tumor necrosis factor alpha converting enzyme
tmTNF-α: transmembrane tumor necrosis factor-α
TNFR1: tumor necrosis factor receptor 1
TNFR2: tumor necrosis factor receptor 2
TNF-α: tumor necrosis factor alpha
TRADD: tumor necrosis factor receptor-associated DD
TRAF1: tumor necrosis factor receptor associate factor-1
TRAF2: tumor necrosis factor receptor associate factor-2
